# Multidimensional decision support by artificial intelligence across the full workflow of endoscopic submucosal dissection for early gastric cancer

**DOI:** 10.3389/fonc.2026.1898921

**Published:** 2026-08-17

**Authors:** Chengyuan Mao, Yichao Zheng, Haifeng Jin

**Affiliations:** 1 The Second Clinical Medical College of Guangxi Medical University, Nanning, Guangxi, China; 2 Department of Gastroenterology, The First Affiliated Hospital of Zhejiang Chinese Medical University, Hangzhou, Zhejiang, China

**Keywords:** artificial intelligence, decision support, early gastric cancer, endoscopic submucosal dissection, whole-process management

## Abstract

Early diagnosis and treatment are essential for improving the prognosis of patients with gastric cancer. Endoscopic submucosal dissection (ESD) is the preferred minimally invasive treatment for early gastric cancer (EGC). Yet the entire diagnostic and therapeutic workflow depends heavily on the physician’s experience and is subject to strong subjectivity, procedural variability, and a high rate of missed diagnoses. Artificial intelligence (AI) offers a new approach to addressing these clinical pain points. This review systematically examines the progress of multidimensional AI decision support across the full ESD workflow for EGC. It evaluates the applicable scenarios and limitations of distinct technical pathways, discusses the feasibility of cross-stage integration of AI systems, and analyses the core barriers to clinical translation, including the scarcity of multicentre standardised datasets, limited model interpretability, and poor integration with clinical workflows. Finally, the review anticipates future directions, such as multimodal data fusion and the combination of edge computing with augmented reality technologies. AI is driving ESD diagnosis and treatment towards greater precision and intelligence. The development of an intelligent clinical decision support system (CDSS) that integrates diagnosis, treatment and follow-up may further empower endoscopists to deliver individualised precision therapy for early gastric cancer.

## 1 Introduction

Gastric cancer remains a major global public health challenge. As one of the five most common cancers worldwide, its mortality rate ranks fourth among all cancers, imposing a heavy disease burden globally (1). Patient survival varies markedly with disease stage: the five-year survival rate for early gastric cancer (EGC) exceeds 90%, whereas for advanced gastric cancer it falls below 30% (2–4). This stark contrast highlights the critical importance of early diagnosis and precise treatment. Endoscopic submucosal dissection (ESD), a minimally invasive technique, has become the preferred treatment for patients with EGC who meet established indications (5, 6).

ESD demands accurate preoperative evaluation, skilled intraoperative manipulation, and rigorous postoperative management. Clinical decision-making has traditionally depended on the endoscopist’s personal experience and subjective synthesis of multimodal findings, leading to limited accuracy, missed critical information, and poor workflow standardisation (7).

AI encompasses traditional machine learning and deep learning, which differ in data-processing paradigms, feature extraction, and interpretability. Both have demonstrated clear clinical value in medical image analysis. For instance, Gong et al. reported a real-time endoscopic AI system with a gastric neoplasm detection rate of 95.6%, while Teramoto et al. achieved 97.0% sensitivity and 99.4% specificity using a cascade deep learning model (8, 9).

This review provides the first systematic evaluation of multidimensional AI decision support across the preoperative, intraoperative, and postoperative phases of ESD for EGC, summarising existing achievements, comparing the strengths and weaknesses of different technical pathways, and exploring strategies for their cascaded cross-stage integration, thereby offering theoretical reference and practical guidance for advancing ESD towards greater precision, safety, and intelligence. This review adopts a comprehensive critical approach. The literature was identified through targeted searches of PubMed and Web of Science for studies published up to early 2026, and organized around the preoperative, intraoperative, and postoperative phases of the ESD pathway.

## 2 Application of AI in preoperative decision support for ESD

### 2.1 Assessment of invasion depth and staging optimisation

Preoperative decision-making, the precise identification of patients suitable for ESD, is foundational to avoiding non-curative resection and unnecessary surgical trauma. AI enables objective, individualised predictions by quantitatively analysing clinical and endoscopic data, helping to refine surgical indication assessment.

Accurate determination of tumour invasion depth is the gold standard for defining ESD indications. Endoscopic ultrasonography (EUS) has traditionally been the primary modality for assessing invasion depth, but its accuracy varies considerably and it depends heavily on operator experience. Deep-learning-based image analysis models have recently emerged as a non-invasive, real-time alternative: Chen et al. (10) distinguished T1a from T1b early gastric cancer using white-light endoscopy alone, significantly outperforming traditional diagnostic methods; Gong et al. (8) achieved 89.2% accuracy for invasion depth prediction; Wu et al. (11) surpassed the average endoscopist in predicting both invasion depth and differentiation status; and the multi-task framework of S. Lee et al. (12) attained 88.4% accuracy for submucosal invasion while concurrently assessing tumour differentiation, lymphovascular invasion, and lymph node metastasis risk.

More objective quantitative metrics have recently emerged. Chen K et al. combined standardised colour metrics with endoscopic morphological features in decision tree and random forest models, improving objectivity over conventional subjective assessment by measuring colour differences between cancerous and surrounding normal mucosa (13). Tao et al. developed a hybrid CT radiomics and deep learning model for relatively precise invasion depth and T-stage evaluation (14).

AI models show considerable promise but are affected by lesion complexity, comorbidities, and single-centre designs that limit generalisability. Different technical pathways carry distinct trade-offs. Endoscopic-image-based deep learning offers real-time, non-invasive assessment but degrades with mucus, bleeding, or insufficient insufflation. CT radiomics provides whole-tumour morphological information complementary to endoscopic assessment but has limited spatial resolution for fine mucosal invasion differences and entails radiation exposure, precluding routine screening. Hybrid multimodal models, though theoretically superior, face greater complexity in data alignment and training.

From the perspective of model development and validation, Chen et al. (10) relied on single-centre data, whereas Gong et al. (8), despite performing external validation, used validation cohorts from the same country. Such differences in dataset composition may affect model generalisability. Some studies employed traditional CNN architectures; S. Lee et al. (12) adopted a multi-task learning framework. These architectural choices also contribute to performance variation. Moreover, inconsistent evaluation standards hinder cross-study comparison. Notably, existing research generally lacks interpretability analysis of the model decisions, which limits acceptability in high-risk clinical settings. Federated learning frameworks could mitigate single-centre limitations by enabling privacy-preserving multi-institutional training without centralized data pooling (15). Incorporating *post-hoc* interpretability methods such as Grad-CAM (16) or SHAP (17) would make model predictions more transparent to clinicians, while standardized benchmarks and reporting guidelines would facilitate rigorous cross-study comparison (18).

### 2.2 Outcome prediction of curative resection and indication assessment

EGC carries a risk of non-curative resection after ESD, particularly in cases meeting expanded indications such as undifferentiated-type carcinoma. The machine learning model established by Bang et al. integrates patient age, lesion size, location, and morphology to predict the likelihood of curative resection for undifferentiated EGC, achieving an external validation accuracy of 89.8% (7). The risk scoring system developed by Kim et al. stratifies patients to predict the need for additional gastrectomy after non-curative ESD (19).

Expanded ESD indications remain controversial due to preoperative–postoperative diagnostic inconsistency and insufficient long-term follow-up data (20). In this context, AI prediction models are particularly salient: S. Lee et al. comprehensively predicted multiple pathological features including undifferentiated histology (12), and Kato M et al. predicted lymph node metastasis risk in patients not meeting endoscopic curability criteria (21). Such models integrate multidimensional patient information for more individualised risk predictions than single-indicator assessments. Future fusion of endoscopic images, pathology, and molecular biomarkers may enable even more precise personalised indication assessments (22–24).

Notably, most preoperative prediction models rely on retrospective data with attendant risks of selection bias and overfitting. Although Bang et al. (7) performed external validation, the cohort was small and confined to South Korea; generalisability to other populations remains unverified. Systematic comparison of different algorithms on shared datasets is lacking. How risk probabilities from current models should be integrated with existing ESD guideline indications awaits prospective clinical testing.

In summary, AI has demonstrated clear value in preoperative ESD decision-making by improving the objectivity and accuracy of invasion-depth assessment and curative-resection prediction. Deep learning models now rival or exceed conventional EUS-based staging, and machine learning classifiers can integrate multidimensional patient data to refine indication triage. Nevertheless, the preoperative AI literature remains dominated by single-centre, retrospective designs with limited interpretability analysis. Future work should prioritize multicentre prospective validation, standardized evaluation protocols, and the integration of explainability techniques to build clinician trust in AI-generated preoperative recommendations.

## 3 Application of AI in intraoperative decision support for ESD

### 3.1 Real-time anatomical structure recognition and surgical navigation

AI-assisted intraoperative decision-support systems are increasingly critical to enhancing the precision and safety of ESD. Within the complex and dynamic environment of the gastric cavity, AI can provide real-time recognition of anatomical structures and spatial localization, establishing a spatial reference frame for the operator. Wu et al. developed a deep convolutional neural network system that detects EGC, classifies gastric locations, and monitors blind spots in real time via a gastric mesh model (25). Yuan et al. reported the AIMED system, distinguishing 27 upper gastrointestinal anatomical locations with 99.40% average accuracy (26) Scheppach et al. achieved high Dice scores for segmenting background tissue, submucosal layer, submucosal vessels, and muscularis propria, with a processing delay of only 0.04 s (27).

Performance differences in anatomical structure recognition arise primarily from variations in task definition and evaluation granularity. Wu et al. (25) focused on coarse-grained classification of gastric regions, whereas Scheppach et al. (27) performed pixel-level fine segmentation, which is technically more demanding but clinically more informative. Although the AIMED system by Yuan et al. (26) performed admirably in conventional gastroscopy scenarios, its accuracy may degrade during ESD owing to bleeding, mucus, and instrument obstruction. Systematic evaluation of model robustness under real surgical conditions remains an open research question.

Nevertheless, these technologies are essential for standardizing endoscopic workflows and reducing the learning curve for junior physicians. Looking ahead, the integration of augmented reality (AR), which overlays AI-recognized anatomical structures and lesion information directly onto the operator’s endoscopic view, promises to deliver more intuitive and precise intraoperative real-time navigation (28).

### 3.2 Real-time lesion detection and classification

AI-assisted CDSS has demonstrated substantial efficacy in the real-time detection and classification of lesions during ESD, enabling automated identification and benign/malignant differentiation, thereby addressing the two fundamental clinical questions of whether a lesion exists and what its nature is.

A meta-analysis by Jiang et al. of 16 studies confirmed favourable diagnostic performance for AI-assisted EGC detection (29); Teramoto et al. proposed a cascade model that first classifies images and then segments only cancerous ones, reducing unnecessary computation and false positives (9); and Jin et al. built a Mask R-CNN-based detection model achieving pixel-level segmentation while meeting real-time latency requirements (30).

El Asmar et al. reported that computer-aided detection (CADe) systems have outperformed junior physicians in reducing missed diagnoses of EGC and predicting invasion depth, and in some scenarios match expert performance (31); Lee et al. further validated CADe utility in upper gastrointestinal tumour examination (32). Gong et al. described a deep-learning-based CDSS excelling in both lesion detection rate and a four-class classification task (advanced gastric cancer, EGC, dysplasia, non-neoplastic lesion) (8); and Mask R-CNN-based models provide high-precision detection, offering a visual basis for intraoperative boundary delineation (30).

However, real-world performance faces multiple challenges. Most studies evaluated models on static images, whereas actual endoscopy involves motion blur, mucus, and bleeding; robustness in dynamic video streams remains unverified. All 16 studies in Jiang et al.’s meta-analysis (29) were retrospective, introducing potential image-selection bias. The cascade model of Teramoto et al. (9), while reducing false positives, was validated on single-centre data without multi-centre generalisability assessment.

### 3.3 Real-time boundary recognition and augmented reality navigation

Precise delineation of lesion boundaries is essential to ensuring complete resection while minimizing trauma to healthy tissue. This can be achieved by overlaying AI-recognized boundary contours onto real-time endoscopic images in an AR fashion, providing the operator with intuitive visual navigation.

Deep-learning-based boundary recognition has made substantial progress in recent years. Mao et al. constructed a fused CNN3 model that achieved pixel-level segmentation of EGC boundaries, with recognition accuracy that did not differ significantly from that of senior endoscopists (33). Park et al. developed a patient-specific virtual 3D surgical navigation system capable of precise reconstruction of peri-gastric anatomy and vascular boundaries, together with intraoperative visual field fusion (34).

Boundary recognition technology still faces several technical bottlenecks. The margins of EGC often appear vague and exhibit gradual transitions under conventional white-light endoscopy, lacking clear morphological demarcations. The visual presentation of lesion boundaries also varies across different endoscopic imaging modes, necessitating large volumes of training data. Furthermore, real-time boundary recognition demands extremely low computational latency, yet existing studies have not reported latency data for real-time video-stream processing. Current boundary recognition models predominantly use architectures such as U-Net and DeepLab; the receptive field size and loss-function selection in these models can affect the preservation of boundary detail, and systematic comparisons in the literature are lacking. Domain adaptation techniques could reduce the dependency on large modality-specific training sets by transferring representations across imaging modes (35). Boundary-aware loss functions, such as a combined Dice and boundary loss — may improve the delineation of vague lesion margins (36). For real-time deployment, model pruning, quantization, and edge-computing strategies could bring inference latency within clinically acceptable thresholds; systematic reporting of these metrics would enable meaningful comparison across architectures.

In summary, intraoperative AI systems are progressing from proof-of-concept demonstrations toward clinically viable real-time assistance tools. Anatomical structure recognition, lesion detection and classification, and boundary delineation each address a distinct intraoperative need, and their convergence with augmented reality visualization promises intuitive surgical navigation. However, robust performance under real surgical conditions — with bleeding, mucus, instrument artifacts, and rapid camera motion — remains largely unproven. Systematic latency benchmarking, cross-modality domain adaptation, and prospective intraoperative validation studies are essential next steps toward translating these technologies into the endoscopy suite.

## 4 Application of AI in postoperative decision support for ESD

### 4.1 Complication monitoring and early warning systems

Post-ESD complications can substantially impair patient recovery. AI processes high-dimensional non-linear relationships among diverse factors to achieve refined, individualized risk stratification, shifting the paradigm from passive response to active prevention.

Na et al. developed deep-learning and clinical risk models that effectively stratifying patients into low-, medium-, and high-risk groups (37). The BEST-J scoring system incorporates ten key variables, including antithrombotic drug use and discontinuation, to categorise patients into four bleeding-risk levels (38) The CatBoost model of Maruyama et al. achieved an AUC of 0.80 in predicting 28-day post-ESD bleeding, significantly outperforming traditional logistic regression (AUC = 0.71) (39). Looking ahead, integrating real-time intraoperative data (heart rate, blood pressure, and procedure duration) into these models via Transformer-based temporal architectures could enable dynamic risk updating during surgery, shifting from static preoperative prediction to intraoperative–postoperative joint surveillance.

Beyond bleeding, AI has also been explored for predicting other ESD complications. Park et al. constructed a machine-learning-based risk scoring system for aspiration pneumonia after gastric ESD, with reasonably good predictive performance (40) Such models enable clinicians to implement targeted interventions based on predicted risk levels, reducing complication rates and improving safety (41).

Postoperative complication prediction models face several shared challenges. Most draw on preoperatively or intraoperatively available variables without incorporating real-time intraoperative information for dynamic risk updating. The CatBoost model of Maruyama et al. (39)outperforms logistic regression on class-imbalanced data, yet its greater hyperparameter-tuning complexity impedes clinical deployment. More fundamentally, how model-generated risk probabilities should guide individualised interventions remains untested in prospective studies.

In summary, AI-based postoperative risk stratification represents a shift from uniform follow-up protocols toward individualized surveillance. Models for bleeding, aspiration pneumonia, and recurrence prediction have shown promising discrimination, particularly when using ensemble or gradient-boosting approaches that capture non-linear risk factor interactions. Yet the evidence base remains narrow: most models derive from single-centre cohorts, lack dynamic updating with intraoperative data, and have not been tested in prospective interventional trials. Closing the loop between risk prediction and clinical action — by embedding these models into automated early-warning systems with defined intervention thresholds — will be critical to realizing their clinical impact.

### 4.2 Gastric cancer recurrence risk prediction models

AI has shown potential in predicting long-term tumour recurrence and distant metastasis after ESD, although the evidence base remains limited. One study comparing seven machine learning algorithms for predicting distant metastasis in T1-stage gastric cancer found that a random forest model achieved the highest AUC of 0.941 (42), suggesting that machine learning can quantitatively identify patients at elevated recurrence risk who may need intensified surveillance or additional treatment.

This research remains at an early stage: existing models rely on retrospective cohorts without prospective validation, the extremely low incidence of distant metastasis in T1-stage gastric cancer raises overfitting risk with small samples, and why random forest outperformed other algorithms was not analysed in depth. The optimal decision threshold for balancing sensitivity and specificity across clinical scenarios remains an open question.

In summary, AI-based recurrence prediction after ESD remains at an early stage. Although machine learning models have demonstrated strong discriminatory performance in single studies, prospective validation is lacking, and the extremely low event rate of distant metastasis in T1-stage gastric cancer makes rigorous model evaluation challenging. Future work should focus on multicentre cohorts with longitudinal follow-up to generate the evidence needed for clinical deployment.

## 5 Integration pathways of whole-process AI systems and construction of an integrated decision system

Although AI applications at each stage have made considerable progress, existing systems largely operate in isolation, with little cross-stage data flow or decision coordination. Building an integrated CDSS that spans the full ESD workflow requires systematic integration across three dimensions: the data layer, the algorithm layer and the application layer.

At the data layer, whole-process integration depends on information exchange among preoperative endoscopic images, intraoperative surgical videos and postoperative pathology and follow-up records, together with a standardised multimodal data-sharing mechanism. Preoperative endoscopic images and invasion-depth predictions can serve as input priors for the intraoperative navigation system. Meanwhile, intraoperatively recorded surgical timestamps and operational parameters should be annotated in correlation with postoperative pathology findings, forming a complete data chain that links the preoperative, intraoperative and postoperative phases.

At the algorithm layer, an integrated decision system calls for cross-stage joint optimisation strategies. In intraoperative boundary recognition, for instance, the lesion-invasion probability heatmap produced by a preoperative depth-prediction model can be used as a prior attention map for the intraoperative segmentation network. This would enable the model to increase boundary-recognition sensitivity in regions predicted to have deeper invasion, thereby creating an information cascade from preoperative evaluation to intraoperative navigation. Similarly, operational anomalies detected by an intraoperative workflow-recognition system could trigger dynamic updates to the postoperative complication early-warning model.

At the application layer, the integrated CDSS should be designed with clinical workflow at its centre. An ideal system would offer a unified user interface through which operators can access preoperative evaluation, intraoperative navigation and postoperative early-warning information on the same platform. It should also incorporate adaptive information display that automatically adjusts the content shown according to the surgical stage. Finally, a closed-loop feedback mechanism should channel postoperative follow-up outcomes back into the system, enabling continuous refinement of prediction models at every stage.

In summary, the transition from isolated AI applications to a unified whole-process CDSS demands coordinated innovation across the data, algorithm, and application layers. While the conceptual architecture — multimodal data fusion, cross-stage model cascading, and workflow-centred interface design — is increasingly clear, practical implementations remain nascent. Pilot studies deploying integrated prototypes in real clinical environments, even on a small scale, would generate invaluable evidence on feasibility, workflow compatibility, and user acceptance, paving the way for larger-scale development.

## 6 Technical challenges and future prospects

AI has shown transformative potential for whole-process decision support in ESD for EGC, yet the transition from laboratory research to routine clinical practice faces substantial challenges in data quality, model interpretability, system integration and regulatory compliance.

At present, the principal bottleneck remains data. Building robust, generalisable AI models requires large-scale, standardised clinical datasets annotated with multi-centre heterogeneity. Most available studies, however, are based on single-centre retrospective data, which introduces selection bias; the stability and generalisability of these models urgently need confirmation through large-scale prospective, multi-centre validation (12, 43). An additional concern is that the decision-making process of most deep learning models lacks transparency. Their ‘black-box’ character limits clinical trust and impedes widespread adoption (44)。 Addressing this bottleneck calls for a combination of complementary strategies. Federated learning enables privacy-preserving multi-centre training without centralized data pooling (15); synthetic data augmentation and standardized annotation protocols can expand effective dataset size and reduce inter-centre labelling variability (45); and prospective registry-based validation frameworks would provide the real-world evidence required to confirm model stability and generalisability across diverse clinical settings (18).

A practical roadmap for overcoming these barriers should proceed along four parallel tracks. First, establishing multicentre data consortia with standardized annotation protocols and federated learning infrastructure (15) would address the data bottleneck while respecting privacy regulations. Second, integrating *post-hoc* explainability methods [e.g., Grad-CAM (16), SHAP (17)] into model development pipelines and subjecting AI recommendations to prospective clinical audit would build the trust required for high-stakes decisions. Third, human–computer interaction research should systematically evaluate interface designs, alert strategies, and cognitive-load metrics to define best practices for AI integration into endoscopic workflows. Fourth, regulatory engagement should begin early in the development cycle, with iterative dialogue between developers, clinicians, and regulatory bodies to align technical validation with evidentiary standards for medical device approval. Progress on these four tracks, pursued concurrently rather than sequentially, would substantially accelerate the responsible translation of AI-enabled decision support from the laboratory to the endoscopy suite.

Deep integration of AI with existing endoscopic workflows presents numerous challenges. Poorly designed interaction, such as delayed feedback or frequent non-specific alarms, may increase cognitive load and induce ‘alarm fatigue’ (46) Future research should shift from algorithmic accuracy alone toward optimising human–machine collaboration and establishing standardised clinical validation protocols (47). Attention-guided user interface design and adaptive alert prioritization could reduce cognitive burden by presenting only clinically relevant, temporally appropriate notifications. Prospective randomized trials comparing AI-assisted and conventional workflows will be essential to establish whether these systems improve clinical outcomes rather than merely adding to the endoscopist’s workload.

Clinical deployment of AI must align closely with current ESD treatment guidelines. AI-generated outputs can supplement guideline recommendations with a quantitative basis for individualised decision-making. Widespread implementation, however, encounters economic and accessibility barriers: deployment costs may restrict availability in resource-limited settings, potentially widening the digital divide.

Looking ahead, AI research will evolve toward whole-process integration and deeper technological convergence. An intelligent CDSS covering the entire diagnostic and therapeutic pathway, fusing endoscopic images, pathological slides, genomic data, and clinical texts, could break the information silos that currently separate individual treatment stages, delivering coherent decision support from preoperative evaluation through to postoperative follow-up (48, 49).

Edge computing addresses real-time latency constraints through localised model deployment, improving response speed and stability (50). Augmented reality can overlay virtual anatomical information onto the endoscopic view for intuitive intraoperative navigation (51), while large language models open new possibilities for interactive querying and explanation of decision logic (52).

In future clinical practice, AI will function as an auxiliary decision-making tool that refines treatment strategies through human–machine collaboration, moving endoscopic treatment of EGC toward greater precision and intelligence.

In summary, the translational barriers to AI-enabled ESD decision support are increasingly well characterized, spanning data scarcity, model opacity, workflow integration, and regulatory uncertainty. The four-track roadmap outlined above provides a structured approach to overcoming these barriers, but its success depends on coordinated action across institutions, disciplines, and regulatory bodies. Near-term priorities include launching multicentre federated learning consortia, embedding explainability into model development, and establishing prospective validation frameworks that generate the high-grade evidence required for clinical adoption.

## 7 Conclusion

This review has surveyed the current state of AI-enabled decision support across the full pathway of endoscopic submucosal dissection for early gastric cancer — from preoperative evaluation through intraoperative navigation to postoperative management — and has examined the technical architectures, clinical applications, and limitations of systems at each stage. The evidence indicates that AI has progressed beyond a single-purpose diagnostic aid towards a multidimensional support framework spanning the entire care continuum. Preoperatively, deep mining of imaging and clinical data sharpens screening accuracy and invasion-depth assessment. Intraoperatively, real-time recognition and navigation technologies compensate for visual blind spots and help maintain procedural safety. Postoperatively, risk-stratification models enable follow-up strategies to become more individualized and, in certain scenarios, shift towards active prevention.

Yet the translation of these capabilities into routine clinical practice remains constrained by persistent bottlenecks in data quality, algorithmic transparency, and workflow integration. Most studies to date are single-centre and retrospective in design; the generalizability and robustness of AI models under real-world conditions therefore lack high-grade evidence. Moreover, the inherent ‘black-box’ nature of deep learning algorithms continues to impede the development of clinical trust — a prerequisite for adoption in high-stakes decisions.

Addressing these barriers will require the construction of standardised, multimodal datasets that span the preoperative, intraoperative and postoperative phases, alongside multicentre prospective validation of AI systems. Progress also depends on advances in interpretable AI, on the establishment of information-transfer mechanisms that link preoperative prediction with intraoperative navigation and postoperative surveillance, and on privacy-preserving technologies that allow cross-institutional model generalisation without compromising data security. Through deeper multimodal data fusion and human-centred interface design, AI systems can be integrated more organically into clinical practice — ultimately supporting an intelligent closed-loop management platform that unifies diagnosis, treatment and follow-up. The four-track roadmap proposed above, spanning federated data consortia, explainability integration, human–computer interaction research, and early regulatory engagement, offers a practical framework for coordinating these efforts across institutions and disciplines.
